# Supplementary material for: Detecting quantum critical points in the t-t′ Fermi-Hubbard model via complex network theory
Source: Sci Rep. 2020 Nov 24;10:20470. doi: 10.1038/s41598-020-77513-0 (PMC7686386; doi:10.1038/s41598-020-77513-0)
Supplement: Supplementary file 1 — Supplementary Information. [file 41598_2020_77513_MOESM1_ESM.pdf]

Supplemental Material

to the article "Detecting quantum critical points in the $t - t'$ Fermi-Hubbard model via complex network theory"

by Andrey A. Bagrov, Mikhail Danilov, Sergey Brener, Malte Harland, Alexander I. Lichtenstein & Mikhail I. Katsnelson

In this Supplemental Material, we provide results for the dependence of complex network measures on Coulomb repulsion U at different values of next-neighbor hopping t'. We also show figures for the periodic boundary conditions for the $(6,6)$ sector, - the only one where the transition point is evident for periodic b.c.

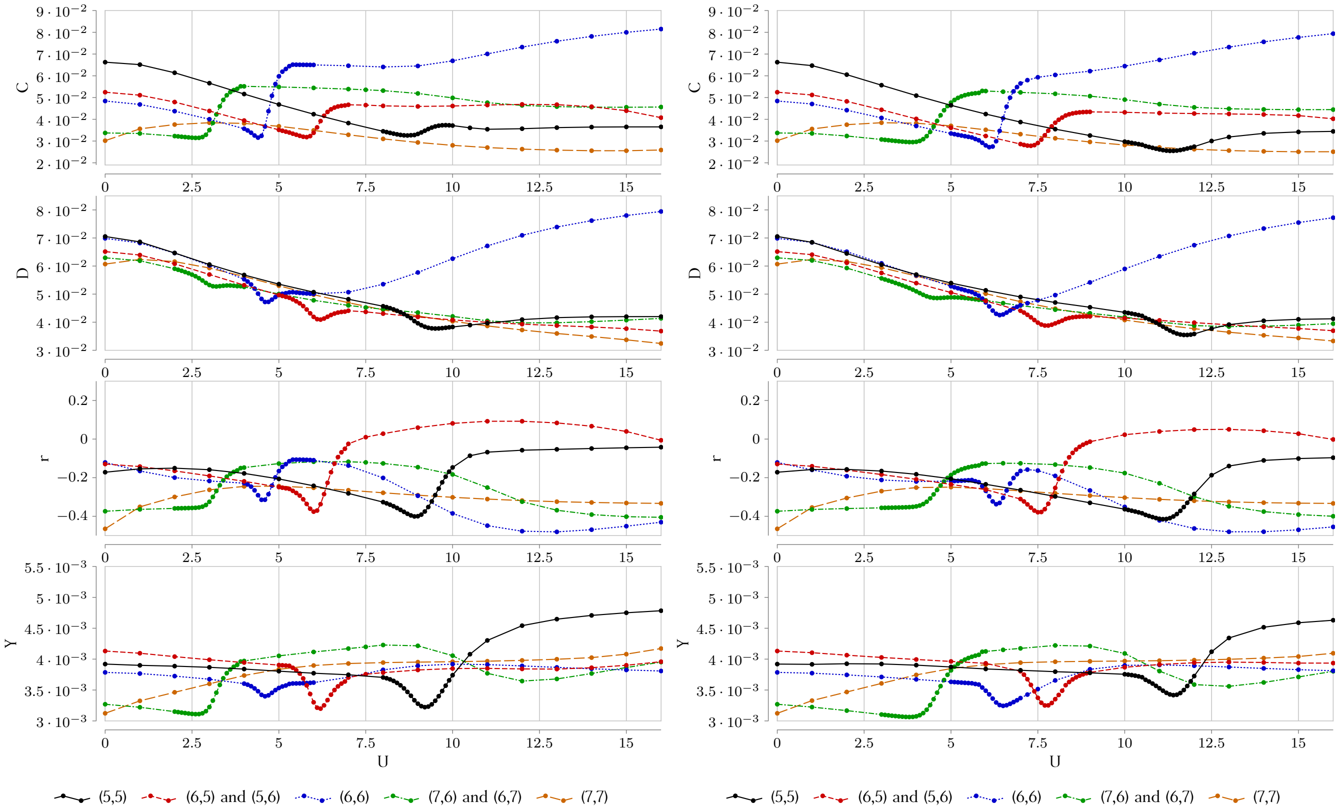

FIG. S1. Characteristics of the mutual information complex network, – clustering C, density D, Pearson correlation r between neighboring sites in the middle of the 4-by-4 plaquette, and disparity Y of a site in the middle of the plaquette, – as functions of the on-site Coulomb repulsion U computed in different sectors for non-periodic boundary conditions. The hopping at $t' = -0.35$ (left) and $t' = -0.32$ (right), the inverse temperature is $\beta = 100$.

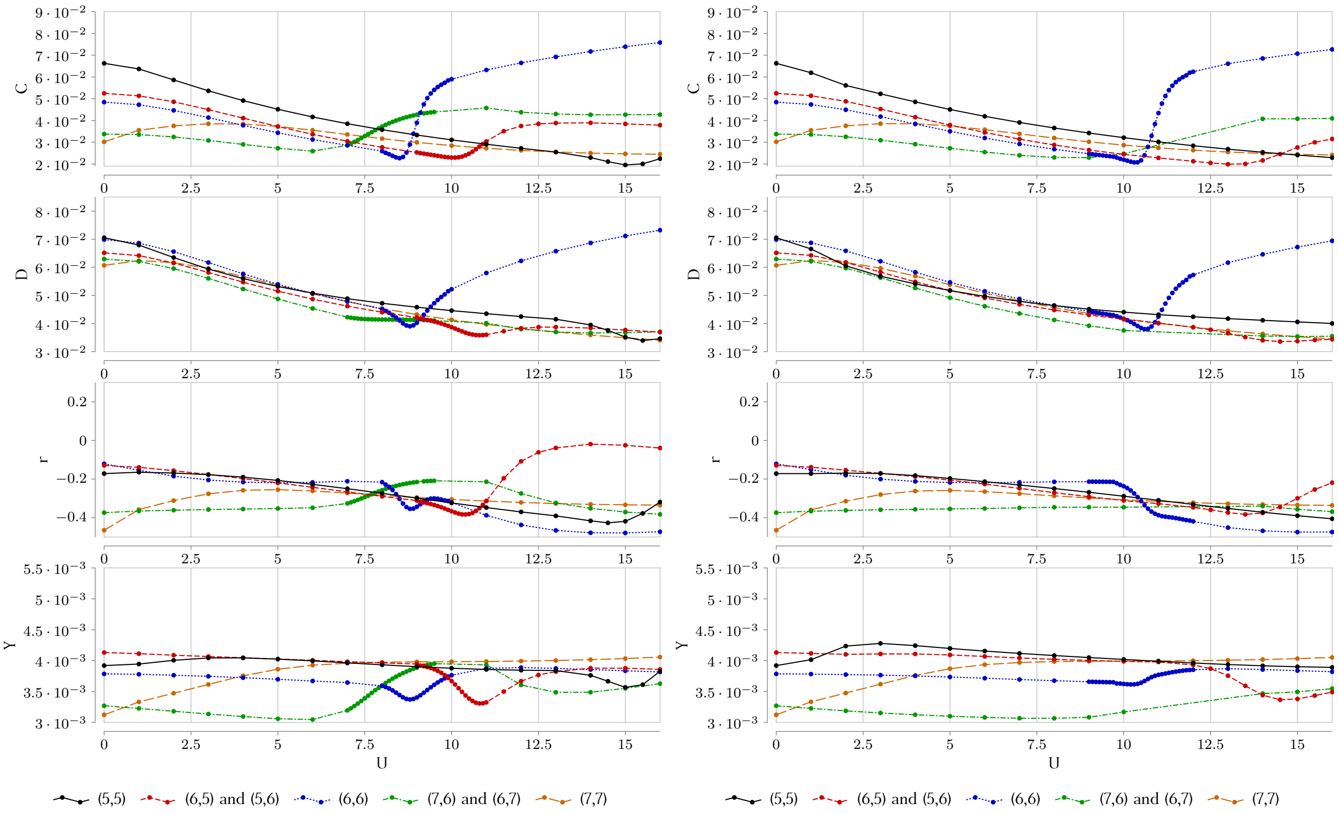

FIG. S2. Characteristics of the mutual information complex network, – clustering C, density D, Pearson correlation r between neighboring sites in the middle of the 4-by-4 plaquette, and disparity Y of a site in the middle of the plaquette, – as functions of the on-site Coulomb repulsion U computed in different sectors for non-periodic boundary conditions. The hopping at $t' = -0.28$ (left) and $t' = -0.25$ (right), the inverse temperature is $\beta = 100$.

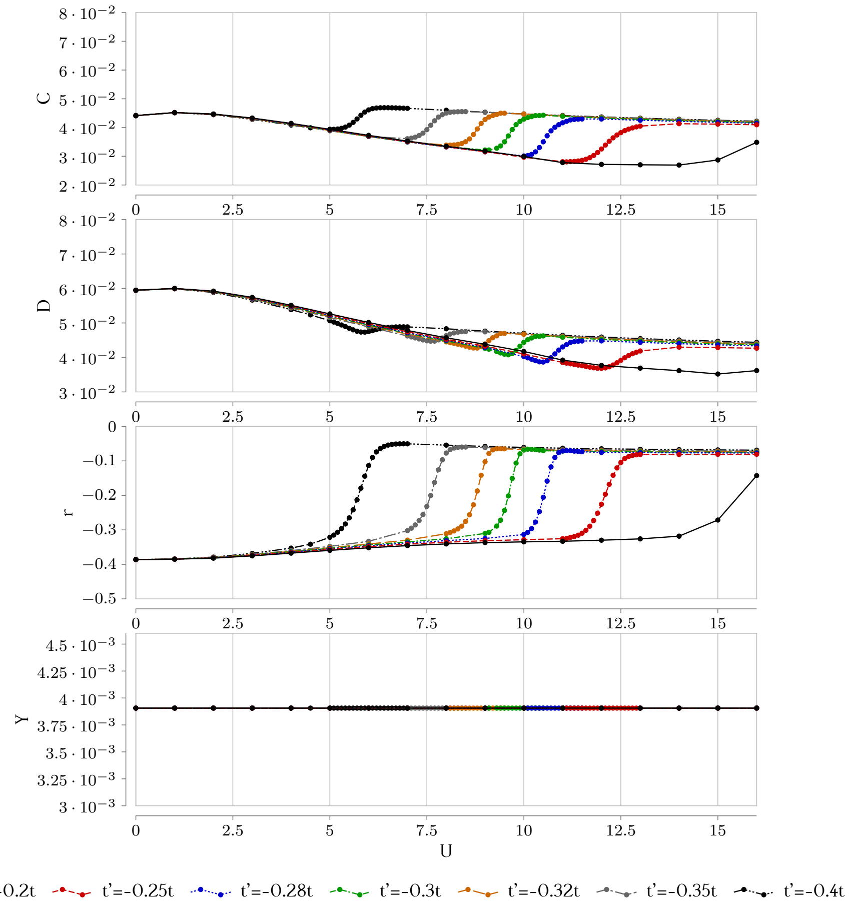

FIG. S3. Characteristics of the mutual information complex network, – clustering C, density D, Pearson correlation r between neighboring sites in the middle of the 4-by-4 plaquette, and disparity Y of a site in the middle of the plaquette, – as functions of the on-site Coulomb repulsion U computed in the (6,6) sector for periodic boundary conditions. It is the only sector where mild features of the phase transition survive upon changing the boundary conditions.

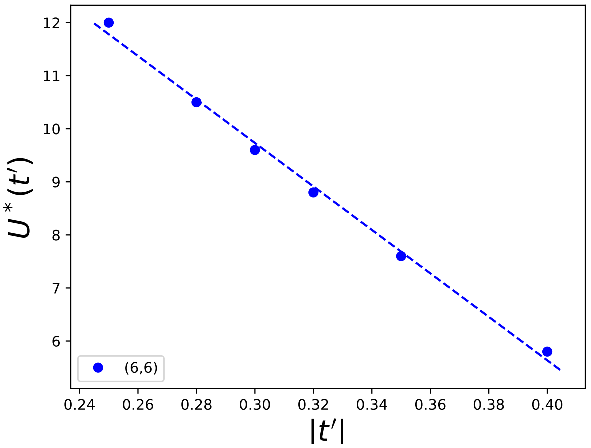

FIG. S4. Dependence of the critical Coulomb repulsion U^* on the next-neighbor hopping t', as the latter is varied in the range $t' \in [-0.4, -0.25]$ for periodic boundary conditions at inverse temperature $\beta = 100$, sector $(6,6)$. The points correspond to locations of disparity minimum (which also coincide with the points where the second derivative of clustering and Pearson coefficients with respect to U changes sign).
